# *Astragalus* Polysaccharide Improves Palmitate-Induced Insulin Resistance by Inhibiting PTP1B and NF-κB in C2C12 Myotubes

**DOI:** 10.3390/molecules17067083

**Published:** 2012-06-11

**Authors:** Ming Zhao, Zhao-Feng Zhang, Ye Ding, Jun-Bo Wang, Yong Li

**Affiliations:** Department of Nutrition and Food Hygiene, School of Public Health, Peking University, Beijing 100191, China

**Keywords:** insulin resistance, NF-κB, PTP1B, *Astragalus* polysaccharide

## Abstract

We investigated the effects of *Astragalus* polysaccharide (APS) on palmitate-induced insulin resistance in C2C12 skeletal muscle myotubes. Palmitate-reduced glucose uptake was restored by APS. APS prevented palmitate-induced C2C12 myotubes from impaired insulin signaling by inhibiting Ser307 phosphorylation of insulin receptor substrate-1 (IRS-1) and increasing Ser473 phosphorylation of Akt. Moreover, the increases in protein-tyrosine phosphatase-1B (PTP1B) protein level and NF-κB activation associated with palmitate treatment were also prevented by APS. However the treatment with APS didn’t change AMP-activated protein kinase (AMPK) activation in palmitate-induced myotubes. The results of the present study suggest that *Astragalus* polysaccharide inhibits palmitate-induced insulin resistance in C2C12 myotubes by inhibiting expression of PTP1B and regulating NF-κB but not AMPK pathway.

## 1. Introduction

Metabolic syndrome (MS) refers to the clustering of obesity, dyslipidemia, diabetes mellitus or impaired glucose tolerance, and hypertension. MS is closely associated with insulin resistance. The plasma concentration of free fatty acid (FFA) in subjects with MS is frequently elevated [1]. With the worldwide escalating risk of MS, investigators have paid high attention to its prevention and treatment. As an important ingredients of “Qi tonifying” prescription, *Astragalus membranaceus* has been used in Chinese herbal prescriptions for more than 2,000 years. It has been proved that *Astragalus* contains active ingredients including *Astragalus* polysaccharides, flavonoids, astragalosides I-VII, amino acids, and trace elements [2]. Previous studies have shown that APS has antioxidant, anti-hypertensive, immunomodulatory, insulin-sensitizing and hypoglycemic activity, anti-obesity and hypolipidemia effects [3,4,5,6].

AMPK exerts pleiotropic effects on cellular metabolism and has emerged as a therapeutic target for MS [7]. At a molecular level, a complex relationship exists between AMPK and the insulin signaling pathways. For instances, AMPK has been reported to regulate IRS-1 and Akt/PKB, while insulin and Akt have negative impacts on AMPK activation [8]. Previous studies suggested that APS can alleviate glucose toxicity via activation of AMPK in high glucose-treated myotubes which were not proven to be insulin resistant [9]. There remains a question that if APS still acts through AMPK pathway in insulin resistant myotubes induced by palmitate.

PTP1B is widely expressed in insulin-sensitive tissues and acts through dephosphorylating phosphotyrosine residues on insulin receptor and IRS-1. Overexpression of PTP1B in liver and muscle suppresses insulin signals [10,11]. Palmitate has been reported to induce insulin resistance by increasing PTP1B expression in the insulin target tissues [12]. Previous studies *in vivo* have shown that APS enables insulin-sensitizing and hypoglycemic activity probably via deceasing PTP1B expression and activity [5,6]. However it is unclear whether APS has the same effect *in vitro*.

NF-κB plays a critical role in implementing the pathogenesis in a number of inflammatory diseases including type 2 diabetes [13,14,15]. FFA has been shown to activate NF-κB and its nuclear translocation that compromised insulin sensitivity in skeletal muscle cells [16]. Inhibitor of κB kinase (IKK) has been implicated in the mediation of FFA induced insulin resistance [16,17]. Whether APS has an effect on NF-κB pathway in insulin-resistant myotubes remains unknown.

Since the mechanisms responsible for the alleviation of insulin resistance under the high level of FFA still remain unclear, we sought to evaluate the pharmacological effect of APS and to investigate in more detail its mechanism by using an insulin-resistant cell model induced by palmitate.

## 2. Results and Discussion

### 2.1. APS Increased Insulin-Induced Glucose Uptake in Palmitate-Treated C2C12 Myotubes

**Figure 1:**
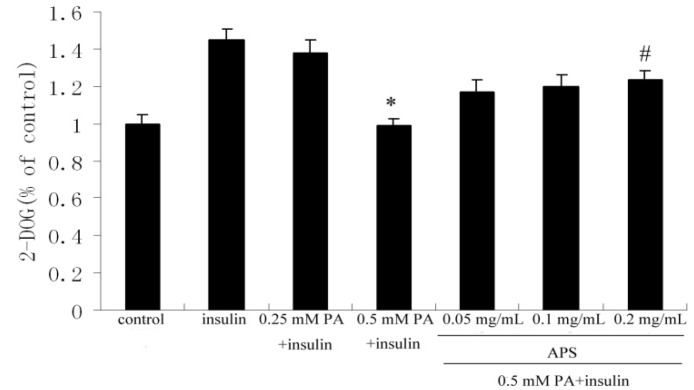
The effect of APS on insulin-stimulated glucose uptake in palmitate-treated C2C12 myotubes. C2C12 myotubes were incubated with either APS or palmitate (0.25 mM or 0.5 mM) or insulin (100 nM) and then assay for 2-DOG uptake as described. Each value is expressed as means ± SD of three determinations. * *p* < 0.05, as compared with insulin control group, ^#^ *p* < 0.05, as compared with 0.5 mM PA group.

To clarify the role of APS in palmitate-induced insulin resistance, glucose uptake was assessed. Treatment with 0.5 mM palmitate (PA) for 12 h lowered stimulatory effect of insulin on glucose uptake by 32% (*p* < 0.05) in comparison with untreated cells. However, subsequently treating with APS for 12 h restored palmitate-reduced glucose uptake in a dose-dependent manner. In the presence of 0.2 mg/mL APS, insulin stimulated glucose uptake was improved by 25% (*p* < 0.05) compared to the cells treated only with 0.5 mM palmitate (Figure 1).

### 2.2. APS Prevented the Inhibition of Insulin Signaling via Suppressing Protein Expression of PTP1B but not via Phosphorylation of AMPK Thr172 in Palmitate-Induced C2C12 Myotubes

**Figure 2:**
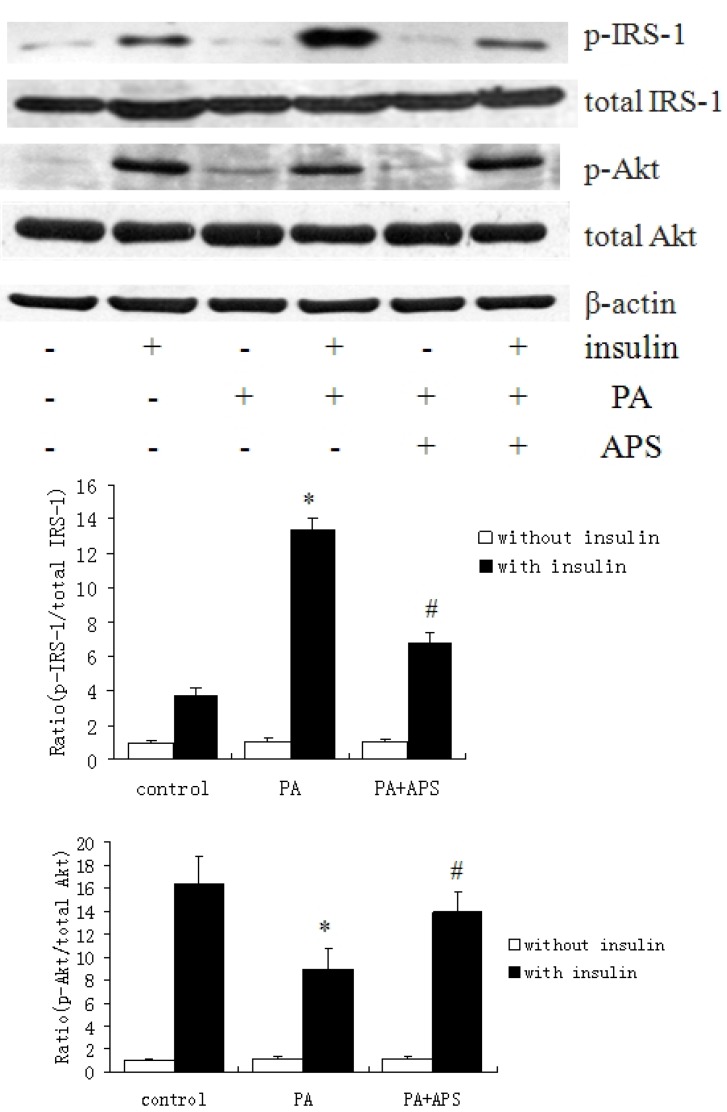
Effects of APS on the palmitate-inhibited insulin signaling pathway in C2C12 myotubes. C2C12 myotubes were incubated for 12 h with 0.5 mM palmitate to induce insulin resistance, subsequently were treated with 0.2 mg/mL APS for 12 h. Before harvesting, the cells were incubated in the presence or absence of 100 nM insulin for 30 min and lysed. Each value is expressed as means ± SD of three determinations. * *p* < 0.05, as compared with insulin control group, ^#^ *p* < 0.05, as compared with PA group in the present of insulin.

To determine whether APS reversed palmitate-induced insulin resistance in C2C12 myotubes by restoring insulin signaling, we examined the phosphorylation of IRS-1 and Akt. We found that palmitate induced IRS-1 Ser307 phosphorylation in the present of insulin, which was significantly reduced by 0.2 mg/mL APS (Figure 2). The treatment with palmitate clearly blocked insulin-induced Ser473 phosphorylation of Akt, which was reversed by the treatment with APS (Figure 2).

**Figure 3:**
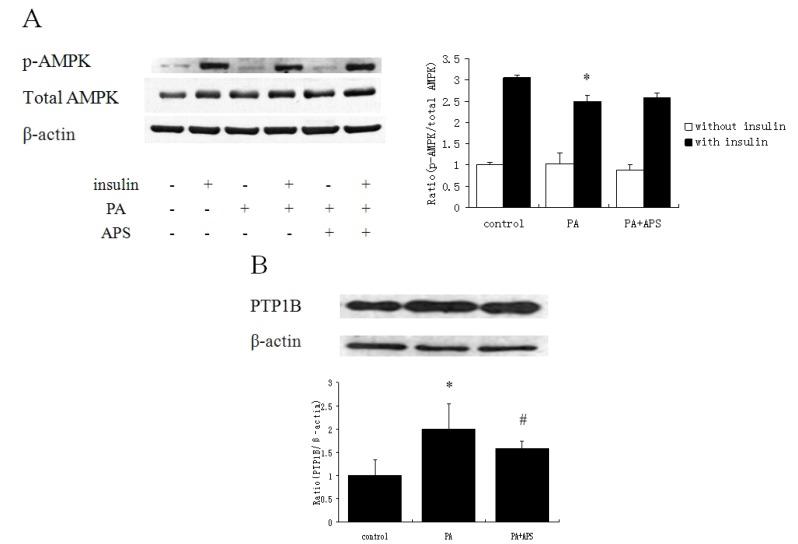
(**A**) The effect of APS on Thr172 phosphorylation status of AMPK in palmitate-induced C2C12 myotubes. C2C12 myotubes were incubated for 12 h with 0.5 mM palmitate to induce insulin resistance, subsequently were treated with 0.2 mg/mL APS for 12 h. Before harvesting, the cells were incubated in the presence or absence of 100 nM insulin for 30 min and lysed. Each value is expressed as means ± SD of three determinations. * *p* < 0.05, as compared with insulin control group. (**B**) The effect of APS on protein level of PTP1B in palmitate-induced C2C12 myotubes. C2C12 myotubes were treated with 0.5 mM PA for 12 h, followed by the treatment with 0.2 mg/mL APS for 12 h. The figure shows representative data gained from the means ± SD of three independent experiments. * *p* < 0.05, as compared with control group, ^#^ *p* < 0.05, as compared with PA group.

To find the factor mediating IRS-1 phosphorylation, we examined the phosphorylation of AMPK in C2C12 myotubes (Figure 3A). PA prominently deceased Thr172 phosphorylation of AMPK. However, treating with APS had no significant improvement on Thr172 phosphorylation. Treating with palmitate provoked increment in PTP1B protein level, which was reversed by APS (Figure 3B).

### 2.3. APS Downregulates NF-κB Signaling Pathway

**Figure 4:**
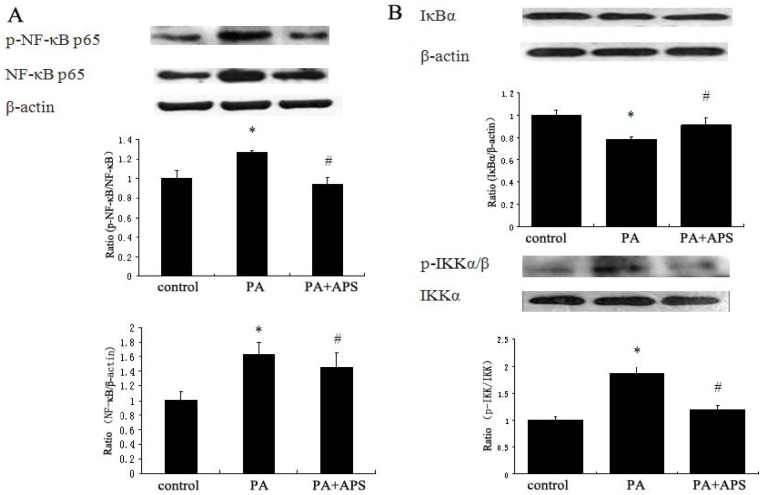
Effects of APS on activation of NF-κB in C2C12 myotubes. C2C12 myotubes were treated with 0.5 mM PA for 12 h, followed by the treatment with 0.2 mg/mL APS for 12 h. (**A**) The effect of APS on phosphorylation and protein expression of NF-κB p65 in palmitate-induced C2C12 myotubes. The densitometry was calculated as phosphorylated NF-κB p65/total NF-κB p65 and NF-κB p65/β-actin. (**B**) The effect of APS on protein level of IκBα and phosphorylation of IKKα/β. The densitometry was calculated as IκBα/β-actin and phosphorylated IKKα/β/ IKKα. The figure shows representative data gained from the means ± SD of three independent experiments. * *p* < 0.05, as compared with control group, ^#^ *p* < 0.05, as compared with PA group.

PA significantly increased the protein expression of NF-κB p65 and its phosphorylation as compared to control, while APS prevented both of them (Figure 4A). APS inhibited the activation of palmitate-induced stress kinases IKKα/β, and restored protein expression of IκBα as compared to PA group (Figure 4B).

### 2.4. Discussion

A key cause of muscle insulin resistance is lipotoxicity. Insulin resistance is commonly associated with obesity and obese-related features, including elevated plasma FFA levels [21]. Skeletal muscle is the major site for insulin-stimulated glucose uptake. The potential effect of APS on insulin-stimulated glucose uptake in C2C12 myotubes exposed to palmitate was investigated in the present study. Our results validate that APS has enhancement on insulin-stimulated glucose uptake in palmitate -induced C2C12 myotubes which is consistent with its functions of lowering blood glucose level and improving insulin resistance in animal experiments [5,6,9,18].

Since previous data have confirmed that APS has hypoglycemic effects in a high-fat diet induced insulin resistant animal model [9], a crucial question herein that remains is how APS can ameliorate the palmitate-induced insulin resistance. Our data suggested that APS abolished insulin resistance by restoring palmitate-inhibited insulin signaling in C2C12 myotubes including the activity of IRS-1 and Akt. Here, our data provided the molecular mechanism how APS can alleviate insulin resistance induced by palmitate at cellular level. Insulin signaling involves a complex signaling cascade downstream of the insulin receptor at the cellular level. AMPK pathway is also a major regulator of glucose transporter 4 (GLUT4) translocation during exercise or in response to some antidiabetic agents such as metformin [22]. We showed that APS has little effect on the decreased activation of AMPK induced by palmitate, suggesting that AMPK signaling pathway may not be responsible for the APS stimulation of glucose uptake in palmitate-induced myotubes. However, it is worthy of further study to confirm whether APS affects other phosphorylation site of AMPK, which can also contribute to the activation of AMPK pathway. Studies have shown that pathological concentrations of palmitate may be partly responsible for PTP1B overexpression observed in insulin resistance states [23]. Downregulating the PTP1B led to enhanced sensitivity to insulin action even in the insulin resistant myotubes. In our study, APS plays as a downregulation role of PTP1B protein level in palmitate-induced C2C12 myotubes, which explains how APS upregulated insulin signaling. This result is consistent with previous studies *in vivo*.

NF-κB regulates the expression of proinflammatory and anti-apoptotic genes [24,25]. Studies in rodents and humans have showed that insulin resistance induced by lipid infusion or an obesity-inducing high-fat diet was associated with decreased IκBα protein levels in skeletal muscle, which are suggestive of NF-κB activation [26,27]. Phosphorylation and dephosphorylation of NF-κB is a major regulatory event for NF-κB nuclear translocation following its dissociation from IκBα. APS could suppress the production of TNF-α and IL-1β by LPS stimulated macrophages by inhibiting NF-κB activation [28]. We show that APS inhibits the activation of palmitate-induced stress kinase IKKα/β and restores IκBα. These evidences suggest that APS attenuates palmitate-induced insulin resistance in C2C12 myotubes via inhibiting activation of NF-κB pathway.

## 3. Experimental

### 3.1. Chemicals and Reagents

*Astragalus* polysaccharide of about 98% purity was purchased from Pharmagenesis, Inc. (Redwood City, CA, USA). There was no detectable level of endotoxin (<0.10 endotoxin units/mL) in the APS samples by the assay of Endospecy. The mouse C2C12 myoblast cell line was obtained from ATCC (CRL 1,772, American Types Culture Collection, Manassas, VA, USA). High glucose-DMEM, fetal bovine serum (FBS) and horse serum were from GIBCO (Grand Island, NY, USA). Insulin, 2-DOG, cytochalasin B, fatty acid-free bovine serum albumin (BSA) and palmitate (PA) were purchased from Sigma-Aldrich (St. Louis, MO, USA). IRS-1, phospho-IRS-1 (Ser307), AMPK, phospho-AMPK (Thr172), Akt, phospho-Akt (Ser473), NF-κBp65, phospho-NF-κBp65 (Ser276), IKKα, phospho-IKKα/β (Ser176/180), IκBα, β-actin antibodies, secondary HRP linked antibodies and ECL were from Cell Signal Technology (Beverly, MA, USA). PTP1B antibody was purchased from Bioworld Technology (Louis Park, MN, USA). PA/BSA conjugates were prepared as previously described [19]. Briefly, 20 mmol/L solution of PA in 0.01 mol/L NaOH was incubated at 70 °C for 30 min, and fatty acid soaps were subsequently complexed with 5% fatty acid-free BSA in PBS at a molar ratio of 8:1.

### 3.2. Cell Culture

C2C12 myoblasts were cultured in high glucose-DMEM containing 10% FBS, 2 mM glutamine, 100 unit/mL penicillin, and 100 μg/mL streptomycin. Cells were maintained at 37 °C under 5% CO2 (v/v) in a humidified incubator. Differentiation of myoblasts into myotubes was induced when the cells had achieved 70% confluence by replacing the media with DMEM containing 2% horse serum. Three to five days later, the fully differentiated myotubes were used for the experiments. 

### 3.3. Glucose Uptake Assay

2-DOG uptake in C2C12 myocytes was conducted according to an earlier method described from our laboratory [20]. The assay was initiated via the addition of 2-DOG (25 mM; 10 mCi/mL) to each of the wells for 10 min at 37 °C. The assay was terminated via the addition and subsequent washing of the cells with ice-cold PBS. The cells were lysed in 10% SDS or 50 mM NaOH. Radioactivity was evaluated via scintillation counting of the lysates extracted in SDS, whereas total protein contents were determined in lysates extracted in NaOH via the Bradford procedure (Bio-Rad Laboratories, Richmond, CA, USA). The glucose uptake values were corrected for non-carrier-mediated transport by measuring glucose uptake in the presence of 10 mM cytochalasin B.

### 3.4. Western Blot Analysis

Western blot analysis was performed by following the method described previously from our laboratory [20]. At the end of the experiments, cells were collected and stored at −80 °C until further analysis. Cells were lysed with ice-cold cell lysis buffer. Protein concentration was then determined using BCA protein assay kit. Samples of cell lysates were separated by 10% SDS-PAGE and then transferred onto PVDF membranes. After being placed in blocking buffer, the membranes were incubated with following primary antibodies (1:1,000 dilution): Anti-IRS-1, anti-Akt, anti-AMPK, anti-PTP1B, anti-NF-κBp65, anti-IKKα, anti-IκBα, anti-p-IRS-1, anti-p-Akt, anti-p-AMPK, anti-p-NF-κBp65 and anti-p-IKKα/β. Then, peroxidase-conjugated secondary antibodies were used. The protein bands were visualized by the ECL kit. The intensities of the protein bands were analyzed by Quantity One (Bio-Rad Laboratories, Hercules, CA, USA). β-actin protein was used as the internal control.

### 3.5. Statistical Analysis

All statistical analyses were performed using SPSS 13.0. (SPSS, Chicago, IL, USA). Comparisons among all groups were performed with the one-way analysis of variance (ANOVA) test. Data were expressed as means ± standard deviation. *p* values < 0.05 were considered statistically significant.

## 4. Conclusions

In summary, we have investigated how palmitate preferentially impaired insulin downsignaling and what role APS played to alleviate this insulin resistance state by using palmitate-induced C2C12 myotubes. The present study demonstrates that APS restores palmitate-reduced glucose uptake in skeletal muscle cells, indirectly preventing insulin resistance by inhibiting expression of PTP1B and activation of IKK/NF-κB instead of AMPK pathway. Further experiments directed at the determining the mechanism of APS may lead to the identification of molecular targets for the generation of therapeutic agents useful in the management of insulin resistance disease like diabetes.
